# Supplementary material for: Time-series transcriptome analysis identified differentially expressed genes in broiler chicken infected with mixed Eimeria species
Source: Front Genet. 2022 Aug 8;13:886781. doi: 10.3389/fgene.2022.886781 (PMC9393255; doi:10.3389/fgene.2022.886781)
Supplement: Supplementary file 2 [file DataSheet1.ZIP › 7dpi_GO.Gsea.1625106495923/GOCC_MITOCHONDRIAL_PROTEIN_CONTAINING_COMPLEX.html]

Details for gene set GOCC\_MITOCHONDRIAL\_PROTEIN\_CONTAINING\_COMPLEX[GSEA]

|  || Dataset | TMM\_7dpi\_gct\_format\_7dpi\_gct\_format.Class\_4dpi.cls #PC\_versus\_NC.Class\_4dpi.cls #PC\_versus\_NC\_repos |
| Phenotype | Class\_4dpi.cls#PC\_versus\_NC\_repos |
| Upregulated in class | 1 |
| GeneSet | GOCC\_MITOCHONDRIAL\_PROTEIN\_CONTAINING\_COMPLEX |
| Enrichment Score (ES) | 0.6101088 |
| Normalized Enrichment Score (NES) | 3.348636 |
| Nominal p-value | 0.0 |
| FDR q-value | 0.0 |
| FWER p-Value | 0.0 |
Table: GSEA Results Summary

  

Fig 1: Enrichment plot: GOCC\_MITOCHONDRIAL\_PROTEIN\_CONTAINING\_COMPLEX      
 Profile of the Running ES Score & Positions of GeneSet Members on the Rank Ordered List

  

| SYMBOL | TITLE | RANK IN GENE LIST | RANK METRIC SCORE | RUNNING ES | CORE ENRICHMENT || 1 | TIMM10B | na | 146 | 1.149 | 0.0022 | Yes |
| 2 | DNAJC19 | na | 208 | 1.036 | 0.0102 | Yes |
| 3 | MRPS12 | na | 242 | 0.999 | 0.0200 | Yes |
| 4 | COX5A | na | 300 | 0.942 | 0.0271 | Yes |
| 5 | NDUFA2 | na | 303 | 0.935 | 0.0388 | Yes |
| 6 | NDUFB6 | na | 307 | 0.933 | 0.0503 | Yes |
| 7 | NDUFS5 | na | 308 | 0.932 | 0.0621 | Yes |
| 8 | CHCHD10 | na | 312 | 0.929 | 0.0736 | Yes |
| 9 | MRPS6 | na | 315 | 0.928 | 0.0852 | Yes |
| 10 | TOMM7 | na | 322 | 0.918 | 0.0963 | Yes |
| 11 | NDUFB7 | na | 323 | 0.915 | 0.1079 | Yes |
| 12 | NDUFB2 | na | 348 | 0.885 | 0.1171 | Yes |
| 13 | MRPL30 | na | 351 | 0.883 | 0.1281 | Yes |
| 14 | NDUFAB1 | na | 356 | 0.876 | 0.1388 | Yes |
| 15 | NDUFA7 | na | 371 | 0.857 | 0.1485 | Yes |
| 16 | NDUFC2 | na | 394 | 0.834 | 0.1572 | Yes |
| 17 | UQCR10 | na | 444 | 0.785 | 0.1630 | Yes |
| 18 | COX6A1 | na | 480 | 0.760 | 0.1697 | Yes |
| 19 | UQCRB | na | 487 | 0.753 | 0.1787 | Yes |
| 20 | NDUFA11 | na | 497 | 0.746 | 0.1874 | Yes |
| 21 | MRPL43 | na | 504 | 0.741 | 0.1962 | Yes |
| 22 | SDHAF4 | na | 516 | 0.730 | 0.2045 | Yes |
| 23 | SMDT1 | na | 548 | 0.711 | 0.2109 | Yes |
| 24 | NDUFA13 | na | 557 | 0.708 | 0.2192 | Yes |
| 25 | MRPL23 | na | 564 | 0.704 | 0.2276 | Yes |
| 26 | MRPL12 | na | 579 | 0.694 | 0.2352 | Yes |
| 27 | NDUFS3 | na | 623 | 0.675 | 0.2401 | Yes |
| 28 | MRPL57 | na | 643 | 0.665 | 0.2469 | Yes |
| 29 | NDUFB3 | na | 656 | 0.657 | 0.2542 | Yes |
| 30 | NDUFS8 | na | 661 | 0.653 | 0.2622 | Yes |
| 31 | NDUFS6 | na | 686 | 0.642 | 0.2683 | Yes |
| 32 | MPV17L2 | na | 697 | 0.638 | 0.2755 | Yes |
| 33 | SDHD | na | 708 | 0.632 | 0.2826 | Yes |
| 34 | BCKDHA | na | 733 | 0.621 | 0.2885 | Yes |
| 35 | UQCC3 | na | 735 | 0.620 | 0.2962 | Yes |
| 36 | NDUFA1 | na | 742 | 0.616 | 0.3035 | Yes |
| 37 | MRPL58 | na | 755 | 0.609 | 0.3102 | Yes |
| 38 | MRPL54 | na | 779 | 0.596 | 0.3158 | Yes |
| 39 | UQCRHL | na | 788 | 0.594 | 0.3227 | Yes |
| 40 | NDUFA5 | na | 794 | 0.593 | 0.3298 | Yes |
| 41 | MRPS33 | na | 798 | 0.591 | 0.3370 | Yes |
| 42 | SDHB | na | 840 | 0.575 | 0.3408 | Yes |
| 43 | MRPL35 | na | 853 | 0.571 | 0.3470 | Yes |
| 44 | DNAJC15 | na | 863 | 0.566 | 0.3534 | Yes |
| 45 | MRPL10 | na | 872 | 0.563 | 0.3599 | Yes |
| 46 | MRPL55 | na | 880 | 0.561 | 0.3664 | Yes |
| 47 | MCUB | na | 883 | 0.561 | 0.3733 | Yes |
| 48 | NDUFB5 | na | 902 | 0.554 | 0.3788 | Yes |
| 49 | MRPS16 | na | 950 | 0.538 | 0.3816 | Yes |
| 50 | NDUFV3 | na | 988 | 0.527 | 0.3852 | Yes |
| 51 | NDUFB4 | na | 1015 | 0.517 | 0.3895 | Yes |
| 52 | NDUFA12 | na | 1023 | 0.513 | 0.3954 | Yes |
| 53 | CHCHD3 | na | 1059 | 0.500 | 0.3988 | Yes |
| 54 | PPIF | na | 1060 | 0.500 | 0.4051 | Yes |
| 55 | TIMM10 | na | 1066 | 0.498 | 0.4110 | Yes |
| 56 | NDUFA6 | na | 1079 | 0.492 | 0.4162 | Yes |
| 57 | NDUFA8 | na | 1080 | 0.492 | 0.4225 | Yes |
| 58 | UQCRQ | na | 1103 | 0.484 | 0.4267 | Yes |
| 59 | NDUFS2 | na | 1124 | 0.477 | 0.4311 | Yes |
| 60 | COX4I1 | na | 1125 | 0.476 | 0.4371 | Yes |
| 61 | SDHC | na | 1135 | 0.473 | 0.4423 | Yes |
| 62 | MRPL21 | na | 1141 | 0.472 | 0.4479 | Yes |
| 63 | MTX2 | na | 1196 | 0.457 | 0.4491 | Yes |
| 64 | NDUFB10 | na | 1215 | 0.450 | 0.4533 | Yes |
| 65 | MRPL15 | na | 1217 | 0.450 | 0.4589 | Yes |
| 66 | TOMM6 | na | 1262 | 0.438 | 0.4607 | Yes |
| 67 | UQCRFS1 | na | 1263 | 0.437 | 0.4662 | Yes |
| 68 | MRPS10 | na | 1264 | 0.437 | 0.4718 | Yes |
| 69 | TOMM20 | na | 1266 | 0.436 | 0.4772 | Yes |
| 70 | ATP5PB | na | 1267 | 0.436 | 0.4827 | Yes |
| 71 | MRPS28 | na | 1280 | 0.432 | 0.4872 | Yes |
| 72 | MRPL40 | na | 1282 | 0.431 | 0.4926 | Yes |
| 73 | NDUFV1 | na | 1307 | 0.428 | 0.4960 | Yes |
| 74 | MRPL45 | na | 1312 | 0.427 | 0.5010 | Yes |
| 75 | NDUFB8 | na | 1330 | 0.423 | 0.5049 | Yes |
| 76 | NDUFS7 | na | 1371 | 0.412 | 0.5068 | Yes |
| 77 | MRPL46 | na | 1380 | 0.409 | 0.5113 | Yes |
| 78 | MRPL28 | na | 1382 | 0.409 | 0.5164 | Yes |
| 79 | NDUFV2 | na | 1395 | 0.408 | 0.5205 | Yes |
| 80 | NSUN4 | na | 1403 | 0.404 | 0.5250 | Yes |
| 81 | TIMM17B | na | 1436 | 0.396 | 0.5273 | Yes |
| 82 | MRPL9 | na | 1437 | 0.396 | 0.5323 | Yes |
| 83 | TIMM50 | na | 1455 | 0.393 | 0.5359 | Yes |
| 84 | MRPL3 | na | 1475 | 0.387 | 0.5392 | Yes |
| 85 | MRPL42 | na | 1521 | 0.375 | 0.5401 | Yes |
| 86 | MRPL19 | na | 1535 | 0.370 | 0.5437 | Yes |
| 87 | MRPL47 | na | 1550 | 0.367 | 0.5472 | Yes |
| 88 | HADHA | na | 1648 | 0.346 | 0.5433 | Yes |
| 89 | MRPS17 | na | 1651 | 0.345 | 0.5475 | Yes |
| 90 | MRPL34 | na | 1672 | 0.339 | 0.5501 | Yes |
| 91 | TIMM22 | na | 1682 | 0.337 | 0.5536 | Yes |
| 92 | MRPS35 | na | 1686 | 0.336 | 0.5576 | Yes |
| 93 | MRPS11 | na | 1692 | 0.335 | 0.5614 | Yes |
| 94 | MRPL33 | na | 1696 | 0.333 | 0.5654 | Yes |
| 95 | UQCRC2 | na | 1700 | 0.332 | 0.5693 | Yes |
| 96 | MRPS18C | na | 1729 | 0.325 | 0.5711 | Yes |
| 97 | MRPL37 | na | 1736 | 0.324 | 0.5747 | Yes |
| 98 | MRPL44 | na | 1799 | 0.307 | 0.5733 | Yes |
| 99 | NDUFB9 | na | 1842 | 0.299 | 0.5736 | Yes |
| 100 | MRPL2 | na | 1843 | 0.299 | 0.5773 | Yes |
| 101 | MRPS21 | na | 1864 | 0.296 | 0.5794 | Yes |
| 102 | MRPL16 | na | 1884 | 0.291 | 0.5815 | Yes |
| 103 | MRPL53 | na | 1984 | 0.273 | 0.5765 | Yes |
| 104 | CHCHD6 | na | 1990 | 0.273 | 0.5796 | Yes |
| 105 | PDHA2 | na | 1992 | 0.272 | 0.5829 | Yes |
| 106 | NDUFB1 | na | 1995 | 0.272 | 0.5862 | Yes |
| 107 | IMMT | na | 2013 | 0.270 | 0.5882 | Yes |
| 108 | TOMM40L | na | 2030 | 0.266 | 0.5902 | Yes |
| 109 | MRPL41 | na | 2082 | 0.257 | 0.5891 | Yes |
| 110 | MRPL24 | na | 2093 | 0.254 | 0.5915 | Yes |
| 111 | SAMM50 | na | 2097 | 0.254 | 0.5945 | Yes |
| 112 | MRPL13 | na | 2109 | 0.251 | 0.5967 | Yes |
| 113 | MRPS9 | na | 2193 | 0.238 | 0.5927 | Yes |
| 114 | BCS1L | na | 2225 | 0.232 | 0.5930 | Yes |
| 115 | MRPL48 | na | 2228 | 0.232 | 0.5958 | Yes |
| 116 | TIMM21 | na | 2269 | 0.226 | 0.5953 | Yes |
| 117 | MRPL14 | na | 2284 | 0.224 | 0.5969 | Yes |
| 118 | SUCLG2 | na | 2297 | 0.223 | 0.5987 | Yes |
| 119 | MRPS14 | na | 2305 | 0.222 | 0.6009 | Yes |
| 120 | UQCRC1 | na | 2326 | 0.217 | 0.6020 | Yes |
| 121 | TIMM13 | na | 2328 | 0.217 | 0.6047 | Yes |
| 122 | HSD17B10 | na | 2339 | 0.216 | 0.6066 | Yes |
| 123 | TIMM9 | na | 2341 | 0.215 | 0.6092 | Yes |
| 124 | MRPS18A | na | 2363 | 0.213 | 0.6101 | Yes |
| 125 | GRPEL1 | na | 2491 | 0.193 | 0.6018 | No |
| 126 | MRPS34 | na | 2496 | 0.193 | 0.6039 | No |
| 127 | MRPL18 | na | 2512 | 0.189 | 0.6050 | No |
| 128 | MRPS5 | na | 2649 | 0.170 | 0.5957 | No |
| 129 | SPG7 | na | 2669 | 0.168 | 0.5962 | No |
| 130 | MRPL39 | na | 2682 | 0.166 | 0.5973 | No |
| 131 | NDUFA4 | na | 2726 | 0.160 | 0.5957 | No |
| 132 | MRPL22 | na | 2747 | 0.158 | 0.5960 | No |
| 133 | MRPL20 | na | 2783 | 0.153 | 0.5950 | No |
| 134 | NDUFA9 | na | 2879 | 0.141 | 0.5887 | No |
| 135 | MRPL38 | na | 2918 | 0.137 | 0.5872 | No |
| 136 | PHB | na | 3009 | 0.125 | 0.5812 | No |
| 137 | DAP3 | na | 3039 | 0.121 | 0.5803 | No |
| 138 | PHB2 | na | 3057 | 0.119 | 0.5803 | No |
| 139 | APOO | na | 3061 | 0.119 | 0.5816 | No |
| 140 | MRPL27 | na | 3152 | 0.107 | 0.5753 | No |
| 141 | MRPS7 | na | 3210 | 0.100 | 0.5718 | No |
| 142 | MRPL51 | na | 3225 | 0.098 | 0.5718 | No |
| 143 | MRPS22 | na | 3240 | 0.095 | 0.5718 | No |
| 144 | MICU1 | na | 3262 | 0.092 | 0.5712 | No |
| 145 | NDUFA10 | na | 3412 | 0.073 | 0.5595 | No |
| 146 | MRPS31 | na | 3424 | 0.071 | 0.5595 | No |
| 147 | POLG | na | 3429 | 0.070 | 0.5601 | No |
| 148 | MRPS26 | na | 3453 | 0.068 | 0.5590 | No |
| 149 | SDHA | na | 3488 | 0.064 | 0.5569 | No |
| 150 | MICU3 | na | 3510 | 0.062 | 0.5559 | No |
| 151 | TRMT10C | na | 3554 | 0.058 | 0.5530 | No |
| 152 | HSPA9 | na | 3575 | 0.055 | 0.5520 | No |
| 153 | DNAJC11 | na | 3589 | 0.054 | 0.5516 | No |
| 154 | PMPCB | na | 3756 | 0.034 | 0.5380 | No |
| 155 | NDUFS1 | na | 3763 | 0.033 | 0.5379 | No |
| 156 | ROMO1 | na | 3798 | 0.028 | 0.5353 | No |
| 157 | MRPL32 | na | 3822 | 0.025 | 0.5337 | No |
| 158 | MRPS27 | na | 3855 | 0.021 | 0.5313 | No |
| 159 | NDUFAF1 | na | 3921 | 0.014 | 0.5260 | No |
| 160 | IMMP1L | na | 3959 | 0.009 | 0.5229 | No |
| 161 | CLPX | na | 4240 | -0.023 | 0.4995 | No |
| 162 | NDUFS4 | na | 4402 | -0.040 | 0.4864 | No |
| 163 | MCU | na | 4548 | -0.056 | 0.4749 | No |
| 164 | AGK | na | 4571 | -0.058 | 0.4737 | No |
| 165 | TIMM23B | na | 4647 | -0.065 | 0.4682 | No |
| 166 | MRPS2 | na | 4677 | -0.068 | 0.4666 | No |
| 167 | MALSU1 | na | 4809 | -0.082 | 0.4566 | No |
| 168 | COX7C | na | 4881 | -0.090 | 0.4517 | No |
| 169 | PNPT1 | na | 5028 | -0.106 | 0.4407 | No |
| 170 | AFG3L2 | na | 5510 | -0.150 | 0.4019 | No |
| 171 | MRPS36 | na | 5609 | -0.160 | 0.3956 | No |
| 172 | PDHB | na | 5684 | -0.168 | 0.3915 | No |
| 173 | MRPL17 | na | 5768 | -0.177 | 0.3867 | No |
| 174 | SLC25A6 | na | 6201 | -0.225 | 0.3530 | No |
| 175 | FOXRED1 | na | 6248 | -0.229 | 0.3520 | No |
| 176 | MRPL50 | na | 6658 | -0.271 | 0.3208 | No |
| 177 | POLRMT | na | 6704 | -0.275 | 0.3204 | No |
| 178 | IMMP2L | na | 6710 | -0.275 | 0.3235 | No |
| 179 | SUPV3L1 | na | 6848 | -0.292 | 0.3156 | No |
| 180 | BCKDHB | na | 6875 | -0.294 | 0.3171 | No |
| 181 | MCCC1 | na | 6896 | -0.297 | 0.3192 | No |
| 182 | MCCC2 | na | 7342 | -0.346 | 0.2859 | No |
| 183 | GRPEL2 | na | 7548 | -0.366 | 0.2732 | No |
| 184 | DLAT | na | 8090 | -0.434 | 0.2329 | No |
| 185 | MFN1 | na | 8095 | -0.435 | 0.2381 | No |
| 186 | MRPS30 | na | 8183 | -0.444 | 0.2363 | No |
| 187 | DBT | na | 8598 | -0.499 | 0.2076 | No |
| 188 | TOMM70 | na | 9089 | -0.563 | 0.1732 | No |
| 189 | MICU2 | na | 9095 | -0.564 | 0.1800 | No |
| 190 | MTX3 | na | 9355 | -0.607 | 0.1657 | No |
| 191 | DNA2 | na | 9396 | -0.615 | 0.1701 | No |
| 192 | APOOL | na | 9563 | -0.640 | 0.1642 | No |
| 193 | PDK1 | na | 10056 | -0.721 | 0.1317 | No |
| 194 | VDAC1 | na | 10060 | -0.721 | 0.1405 | No |
| 195 | NSUN3 | na | 10405 | -0.791 | 0.1214 | No |
| 196 | POLG2 | na | 11536 | -1.131 | 0.0401 | No |
Table: GSEA details [plain text format]

  

Fig 2: GOCC\_MITOCHONDRIAL\_PROTEIN\_CONTAINING\_COMPLEX      
 Blue-Pink O' Gram in the Space of the Analyzed GeneSet

  

Fig 3: GOCC\_MITOCHONDRIAL\_PROTEIN\_CONTAINING\_COMPLEX: Random ES distribution      
 Gene set null distribution of ES for **GOCC\_MITOCHONDRIAL\_PROTEIN\_CONTAINING\_COMPLEX**
